# Atypical Interpersonal Problem-Solving and Resting-state Functional Connectivity in Adolescents with Maltreatment Experience

**DOI:** 10.2174/1570159X22666231002145440

**Published:** 2023-10-05

**Authors:** Mattia I. Gerin, Essi Viding, Vanessa B. Puetz, Diana J.N. Armbruster-Genc, Georgia Rankin, Eamon J. McCrory

**Affiliations:** 1 Division of Psychology and Language Sciences, University College London, London, UK;; 2 Anna Freud National Centre for Children and Families, London, UK

**Keywords:** Childhood maltreatment, childhood trauma, social functioning, interpersonal problem-solving, functional magnetic resonance imaging (fMRI), resting-state functional connectivity (rsFC), default mode network (DMN), medial prefrontal cortex (mPFC)

## Abstract

**Background:**

Childhood maltreatment is associated with altered neurocognitive functioning, which is thought to reflect, in part, adaptation to early adverse environmental experiences. However, we continue to lack a precise mechanistic understanding linking atypical neurocognitive processing with social functioning and psychiatric outcomes following early adversity.

**Objective:**

The present work investigated interpersonal problem-solving, resting-state functional connectivity (rsFC), and mental health symptoms in adolescents with documented maltreatment experience and explored whether altered neural function contributes in part to poorer social functioning.

**Methods:**

Forty adolescents (aged 12-17) with documented experiences of abuse or neglect and a carefully matched group of 42 non-maltreated peers participated in this study that measured task-based interpersonal problem-solving skills and rsFC.

**Results:**

Adolescents with maltreatment experience showed poorer interpersonal problem-solving performance, which partly accounted for their elevated mental health symptoms. Resting-state seed-based analyses revealed that adolescents with maltreatment experience showed a significant increase in rsFC between medial Default Mode Network (DMN) hubs, the medial prefrontal cortex (mPFC), with a posterior cluster, including the posterior cingulate cortex (PCC), precuneus (PCu), retrosplenial cortex (RSC), and lingual gyrus (LG). Moderation analyses revealed that maltreatment-related increased DMN rsFC partly accounted for poorer performance in interpersonal problem-solving.

**Conclusion:**

Poorer interpersonal problem-solving, partly accounted for by atypical coupling between DMN medial hubs, was associated with maltreatment exposure. Interventions tailored to enhance interpersonal problem-solving represents a promising avenue to promote resilience and reduce the likelihood of mental health disorder following maltreatment experience.

## 1 INTRODUCTION

### 1.1 Childhood Maltreatment, Neurocognitive Adaptations, and Social Functioning

A substantial minority of individuals experience childhood maltreatment [1]. This can include acts of commission, such as physical abuse (*e.g*., hitting, kicking, baby-shaking), emotional abuse (*e.g*., belittling, intimidating, blaming, witnessing domestic violence), and sexual abuse (*e.g*., penetrative or non-penetrative sexual acts). Acts of omission include physical neglect (*e.g*., failures to provide adequate clothing, shelter, hygiene, and nutrition that is available) and emotional neglect (*e.g*., denying affective responsiveness and reciprocity or failing to provide access to mental health care). The early experience of abuse and neglect represents one of the most robust environmental predictors of poor mental health and social functioning across the lifespan [2]. Yet, the relationship between early adversity and subsequent psychopathology is not deterministic [3]. A detailed understanding of the mechanisms involved in the etiology of mental health problems can help inform preventative interventions that can promote resilient outcomes and offset risk trajectories before frank disorders emerge [4].

There is a well-established link between maltreatment exposure and neurocognitive changes in domains relevant to social functioning. These include systems that underpin salience/threat detection [5, 6], reward and effort processing [7-10], autobiographical memory [11-14], emotion regulation [15], as well as social attribution and social inference [16, 17]. The theory of “Latent Vulnerability” [18] postulates that these neurocognitive recalibrations following abuse and neglect may confer a short-term advantage in the context of early adversity but, in the longer term, may contribute to the emergence of psychiatric risk by influencing how an individual navigates and shapes their social world [19]. Longitudinal studies have indicated that childhood maltreatment is associated with ‘social thinning’, the reduction in the quality or extent of supportive relationships over time [19]. Many individuals with a history of maltreatment are perceived as less socially competent and likable than their peers and are more likely to experience difficulties in forging and maintaining social bonds [19, 20]. In addition, maltreatment exposure is linked with an increased risk of experiencing more stressful life events, or ‘stress generation’ [21], such as a higher risk of peer rejection, further victimization, and interpersonal conflict [19, 22]. Crucially, social thinning and interpersonal stress generation potentiate the risk of developing mental health symptoms [19-21]. However, we have little understanding of how alterations in neurocognitive processes following maltreatment may influence subsequent (poorly optimized) social functioning [19, 22].

### 1.2 Interpersonal Problem-solving

To address this gap, we investigated interpersonal problem-solving in children and adolescents with maltreatment experiences and carefully matched controls. Interpersonal problem-solving refers to the deliberate process of generating and selecting adaptative strategies in response to everyday interpersonal problems [23]. A key cognitive component of interpersonal problem-solving is means-end thinking, the ability to articulate detailed step-by-step strategies (or Relevant Means) to solve dilemmas or conflicts [24, 25]. Extensive cross-sectional and longitudinal studies have implicated interpersonal problem-solving difficulties with reduced social competence and social adjustment in school, increased occurrence and impact of stressful life events, and higher internalizing and trauma-related psychiatric symptoms [26-33]. Interpersonal problem-solving, including the ability to generate relevant means, is underpinned and influenced by several neurocognitive domains [23, 34], including autobiographical memory [28, 29, 31], mentalizing [35-37], and rumination [28, 38]. Notably, the development of these domains is scaffolded by parenting practices [38-40] and is derailed by childhood abuse and neglect [12, 16, 38]. Collectively, these findings indicate that interpersonal problem-solving, a critical aspect of social functioning and mental health, may be negatively impacted by exposure to maltreatment.

### 1.3 Default Mode Network

Neurocognitive processes that underpin interpersonal problem-solving (*e.g*., autobiographical memory, mentalization) are anchored in the Default Mode Network (DMN) [41]. Constructing mental scenes (for example, during autobiographical memory retrieval) is linked with the preferential engagement of the DMN medial temporal subsystem (*i.e*., the hippocampi and parahippocampal cortex), the angular gyri (AG), precuneus (PCu), and retrosplenial cortex (RSC) [41-43]. Making social inferences, on the other hand, has been linked with the preferential engagement of the cortical midline DMN hubs [41], namely the medial prefrontal cortex (mPFC) and the posterior cingulate cortex (PCC). To the best of our knowledge, no study has directly linked interpersonal problem-solving with a specific pattern of brain activation. Yet, it is likely that the development of the DMN, especially its midline structures (*i.e*., mPFC and PCC), crucial in social-information processing, plays a critical role in the emergence of interpersonal problem-solving skills.

Alterations in DMN connectivity have been postulated to represent a transdiagnostic marker for various mental health problems, difficulties in social cognition, and social functioning [44-47]. For example, increased resting-state functional connectivity (rsFC) of the mPFC with other DMN hubs has consistently been implicated in psychiatric conditions common among individuals exposed to early adversity, such as depression [48], and in social-information processing and neurocognitive domains known to be impacted by maltreatment exposure, such as mentalizing [49] and rumination [50]. Thus, DMN connectivity represents a promising neurobiological marker to understand further the proposed maltreatment-driven alterations in interpersonal problem-solving and poor social outcomes.

### 1.4 Aims and Hypotheses

The first aim of this study was to investigate whether interpersonal means-end thinking is compromised in adolescents who have experienced documented abuse and neglect. In line with prior evidence [19-22], we predicted they would show poorer interpersonal problem-solving performance compared to peers not exposed to early adversity. In line with clinical studies indicating a robust association between interpersonal problem-solving and poor mental health [26-33], we hypothesized that lower interpersonal problem-solving scores among young people exposed to childhood maltreatment would be associated with elevated symptoms. The second aim of this study was to explore maltreatment-associated alterations in DMN connectivity, considering the crucial role of this brain network in social functioning and psychiatric outcomes. Here, we carried out seed-based resting-state analyses on the mPFC. In line with meta-analytic data on depression [48] and rumination [50], we hypothesized that exposure to childhood maltreatment would be associated with a pattern of increased rsFC between the mPFC and other DMN hubs. Finally, given that the DMN is centrally implicated in social functioning, our third aim was to explore if any observed maltreatment-related DMN alterations could partly account for poorer interpersonal problem-solving skills.

## 2 MATERIALS AND METHODS

### 2.1 Participants

Eighty-two adolescents aged 12-17 years participated in this study. Forty had documented maltreatment experiences and were recruited *via* the Social Services Department (the maltreatment group, MT). Forty-two typically developing peers with no prior social service contact (non-maltreatment group, NMT) were recruited *via* schools/advertisements in the community to match the MT group on demographic variables, including age, pubertal status, sex, socioeconomic status, ethnicity, and IQ (Table **1**). A subgroup of 73 (35 MT and 38 NMT) completed a vignette-based interpersonal problem-solving task. Following outlier removal (Supplementary Information, Appendix SI.1), the final subsample consisted of 34 MT and 38 NMT (n = 72) (Table **S1**, Supplementary Information, Appendix SI.4). A subgroup of 63 participants (31 MT and 32 NMT) took part in the resting-state functional magnetic resonance imaging (fMRI) scan. After neuroimaging outlier/movement removal (Supplementary Information, Appendix SI.2), the final sample consisted of 26 MT and 29 NMT (n = 55) (Table **S2**, Supplementary Information, Appendix SI.4). A total of 45 participants (20 MT and 25 NMT) completed both the resting-state scan and interpersonal problem-solving task. For all participants, exclusion criteria included the presence of a pervasive developmental disorder, neurological abnormalities, standard MRI contraindications, and an IQ below 70.

### 2.2 Measures

#### 2.2.1 Maltreatment History

The assessment of maltreatment types (neglect, emotional abuse, sexual abuse, physical abuse, and exposure to domestic violence) was based on social services reports. The severity of each abuse type was rated on a scale from zero (not present) to four, in line with an established measure of maltreatment [51, 52]. In addition, the age of onset and duration of maltreatment by subtype were estimated based on social services’ available documentation. Participants in the MT group (n = 40) were exposed to a mean of 2.6 maltreatment subtypes (SD = 0.9, min. = 1, max. = 4); 10% experienced one, 45% experienced two, 25% experienced three, and 20% experienced four childhood maltreatment subtypes. The most common forms of maltreatment experienced by our sample were emotional abuse, neglect, and exposure to domestic violence (Table **2**). The average onset of maltreatment was three years and 11 months (SD = four years and five months), and the average duration/exposure was seven years and four months (SD = four years and six months).

#### 2.2.2 Cognitive Ability

Verbal and non-verbal intelligence was assessed using two subscales of the Wechsler Abbreviated Scales of Intelligence [53]. Verbal fluency, a measure of executive control, was assessed using the composite score from a phonemic and a semantic fluency task, *i.e*., participants were required to produce in 60 seconds as many words as possible beginning with the letter ‘s’ (phonemic fluency), and from the category ‘animals’ (semantic fluency) [54, 55].

#### 2.2.3 Psychiatric Symptomatology

The Strengths and Difficulties Questionnaire (SDQ) was completed by caregivers to assess general functioning and psychopathology (Table **1**). This is a well-validated measure, with high internal consistency (mean Cronbach α: .73) and good retest reliability (mean correlation: 0.62) [56].

#### 2.2.4 Interpersonal Problem-solving

The Means-end Problem-Solving (MEPS) task measures the ability to generate and articulate step-by-step strategies necessary for achieving a specific goal in problematic interpersonal situations. Participants were presented with four vignettes that described the beginning of a story, the interpersonal problem/conflict, and the end/resolution of the story. Participants were assessed on the number of concrete and effective steps (or ‘Relevant Means’) generated to resolve the interpersonal problem. The MEPS has good internal consistency (typically > 0.80) [24, 57, 58]. Its construct validity has been demonstrated in several studies showing an association between problem-solving skills and psychosocial adjustment [57-60]. Moreover, interpersonal problem-solving performance measured with the MEPS is associated with real-life problem-solving skills [61].

In line with standard scoring procedures implemented in prior studies involving adults and adolescents, we assessed the total number of relevant means, defined as discrete steps taken from the beginning of the story that brings the participant closer to the described problem resolution [24, 28, 30, 62-65]. Relevant means were broken down into ‘Active’ (these are relevant steps initiated by the participant) and ‘Passive’ (these are relevant steps initiated by another person) [64, 65]. Steps lacking detail (*e.g*., “we sorted things out”) with no explanation of how things were resolved and descriptions that were irrelevant to the resolution of the problem were scored as ‘No-Means’ [65]. In line with recent studies, we also included an additional ‘effectiveness’ score. This was rated on a 7-point scale by the experimenter (1 = not at all effective; 7 = extremely effective). A problem-solving strategy is effective if it maximizes positive outcomes and minimizes short- and long-term undesirable consequences, both to oneself and others [23, 28, 64-67]. Vignettes were blindly scored, and a second independent blind rater demonstrated good inter-rater reliability. Additional detailed information about the MEPS, vignettes, and scoring procedures can be found in Supplementary Information (Appendix SI.3).

#### 2.2.5 Resting-state Functional Connectivity Acquisition and Preprocessing

All scans were acquired on a 1.5 Tesla Siemens Avanto MRI scanner using a 32-channel head coil. The resting-state blood oxygen level-dependent (bold) signal was acquired using a whole-brain Echo-Planar Image (EPI) sequence with slices per volume = 36, volumes = 170, voxel size = 3 x 3 x 2 mm, slice thickness = 2 mm, TR = 2880ms, TE = 45 milliseconds, FoV = 192 mm^2^, gap between slices: 1 mm, flip angle: 90, and scanning time = 8 m 10s. Participants looked at a fixation cross throughout the scan. An MRI-compatible camera system provided a face close-up live video of the participants, which was always monitored by two experimenters to ensure that the participants’ eyes remained open during the scanning protocol. The structural scan was obtained using a high-resolution T1-weighted magnetization-prepared rapid gradient-echo sequence (MPRAGE) with slices = 176, slice thickness = 1 mm, gap between slices = 0.5 mm, TE = 2730 ms, TR = 3.57 ms, FoV = 256 mm^2^, and voxel size = 1 x 1 x 1 mm.

The resting-state EPI sequences were pre-processed using CONN v.20.b toolbox [68], Matlab, and SPM12-based software [69]. For each participant, the first three EPI volumes were removed. The remaining volumes were slice-time corrected, realigned, co-registered with their respective anatomical scans, normalized into MNI anatomical space, and segmented into grey matter, white matter, and cerebrospinal fluid (CSF). Functional data were smoothed using a Gaussian kernel of 8mm full-width half maximum (FWHM). Before normalization, ART-based scrubbing [70, 71] was performed to detect outliers by using the most ‘conservative’ (*i.e*., stringent) CONN toolbox option (*i.e*., 0.5 mm FD threshold or 3 s.d. of global bold signal change). After scrubbing, in line with current recommendations, participants with scans shorter than 5 min were removed [70, 71]. Three (9.3%) NMT participants and 5 (16.1%) MT were removed, leaving a total sample of 29 NMT and 26 MT participants with usable neuroimaging data. The number of participants removed as the motion in each group was not statistically significant (*p* = .47). An anatomical component-based noise correction procedure (aCompCor) was used to account for physiological and movement confounds and included noise components from white matter and CSF signal [72, 73], 12 motion parameters [74], the identified outliers scans during scrubbing [70, 71], as well as constant and linear trends [75]. Temporal band-pass filtering was applied to retain only low-frequency fluctuation in the bold signal (between 0.008 Hz and 0.09 Hz). All scans were visually inspected using the quality assurance tools within CONN toolbox.

### 2.3 Data Analysis

#### 2.3.1 Demographics, Cognitive Abilities, and Symptoms

Independent sample t-tests or chi-squared tests, as appropriate, were performed to explore whether the MT and NMT groups differed on demographic characteristics (age, gender, pubertal status, socioeconomic status, ethnicity), cognitive abilities (IQ and verbal fluency), or symptom levels/functioning (SDQ) (Table **1**).

#### 2.3.2 Interpersonal Problem-solving Skills

An independent samples t-test was performed to investigate if MT and NMT groups differed on interpersonal problem-solving skills/means-end thinking (*i.e*., Relevant means total score). A structural equation modeling (SEM) analysis implemented in the R package Lavaan [76] was used to explore if variability in MEPS performance could explain (*i.e*., cross-sectionally mediate) the association between maltreatment status and general psychopathology and functioning (indexed by the SDQ total score).

#### 2.3.3 Resting-state Functional Connectivity Seed-based Analyses

First-level whole-brain seed-based connectivity analyses were performed in CONN toolbox using the mPFC as the seed. All time-series correlation coefficients were Fisher r-to-z transformed. The mPFC seed (coordinates = 1, 55, -3; volume = 10,770 m^3^) was taken from the DMN-specific masks included in the CONN toolbox that was generated from the Human Connectome Project (n = 497) [68, 77]. In second-level analyses, group differences were examined using independent-sample t-tests. In line with recommended neuroimaging whole-brain functional analyses standards [78], the initial height voxel threshold was set as 0.001 uncorrected, and the cluster threshold was then set at 0.05 p-FWE corrected.

Functional connectivity correlation coefficient values were extracted from the region where group differences were observed and used for further analyses. A moderation analysis was performed to explore if group differences in the MEPS were influenced by variability in mPFC rsFC. Post-hoc ROI-to-ROI analyses were also performed to further explore rsFC within the DMN (Supplementary Information for details on the post-hoc ROI-to-ROI analyses - Appendix SI.2).

### 2.4 Procedure

Demographic information (including age, gender, ethnicity, socioeconomic status, and pubertal stage), symptoms questionnaire, and psychometric testing were completed by the participant and one parent/carer. The MRI brain scan and MEPS were performed at the university campus.

## 3 RESULTS

### 3.1 Interpersonal Problem-solving and Childhood Maltreatment

On the MEPS task, we found a large (*i.e*., *d’* = .93) and statistically significant group difference in the relevant means total score (Table **3**). Compared to the NMT group, the MT group generated fewer relevant means. In other words, young people exposed to childhood maltreatment showed poorer interpersonal means-end thinking performance. In particular, the MT group generated fewer active and passive relevant means (Table **3**). Therefore, the composite relevant means total score, rather than the passive and active relevant means subscores, was used in further analyses to reduce the number of multiple comparisons. The effectiveness score also revealed that the MT group generated less effective interpersonal problem-solving strategies (Table **3**). Finally, the number of irrelevant or unspecific information/ steps generated was comparable across groups (Table **3**). Sensitivity analyses revealed that the reported group difference remained significant (t(52) = 3.74, *p* < .001, *d’* = 1.03) after removing participants who met the clinical threshold on the SDQ total score (*i.e*., SDQ total scores equal or above 17; removed MT = 7 and NMT = 4) and had missing SDQ data (removed MT = 4 and NMT = 3).

### 3.2 Interpersonal Problem-solving, Childhood Maltreatment, and Mental Health

**Fig. (1):**
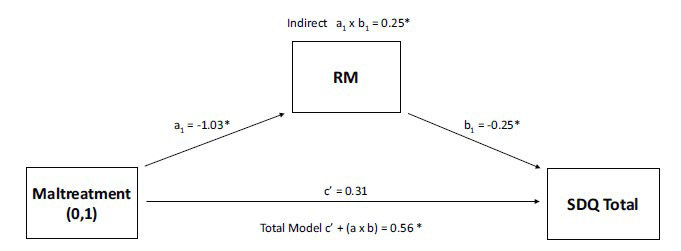
Structural Equation Mediation Model Depicting the Association Between Maltreatment Status, Relevant Means Total Score (RM), and Strength and Difficulties Questionnaire Total Score (SDQ Total). **Note:** This mediation model suggests that greater interpersonal social skills difficulties among adolescents exposed to childhood maltreatment are associated with elevated maltreatment-related mental health symptoms. Coefficient values are standardized; Significance thresholds were measured using bootstrapping; *Statistically significant coefficients.

The relevant means total score cross-sectionally mediates the association between maltreatment status and SDQ total score (Fig. **1**, indirect pathways *a_1_ x b_1_*). In other words, poorer interpersonal problem-solving among young people exposed to childhood maltreatment was associated with reduced mental health and psychosocial functioning.

### 3.3 Resting-state Functional Connectivity and Childhood Maltreatment

**Fig. (2):**
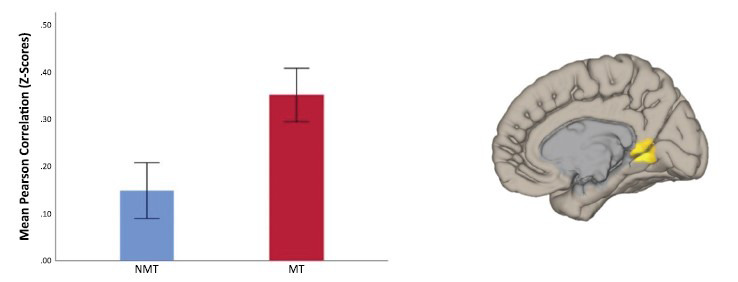
mPFC Seed-Based Resting State Functional Connectivity (rsFC) Differences Between Adolescents with Maltreatment History (MT) and Adolescents with No Maltreatment History (NMT). **Note:** Adolescents exposed to childhood maltreatment (MT) compared to peers not exposed to abuse and neglect (NMT) showed significantly (FWE *p* < .05) increased positive resting-state functional connectivity (rsFC) between the two main cortical midline nodes of the Default Mode Network (DMN): the medial prefrontal cortex (mPFC), which was the seed in this analysis, and a posterior medial cluster, which encompasses the posterior cingulate cortex (PCC), precuneus (PCu), and retrosplenial cortex (RSC). The cluster also included medial portions of the lingual gyrus (LG).

A whole-brain seed-based rsFC map (Fig. **2**) revealed that the MT group, compared to the NMT group, showed significantly greater positive rsFC (p-FWE = 0.028) between the mPFC and a posterior medial cluster (k = 54, peak coordinates = 12, - 51, 03), which encompassed the main posterior DMN midlines structures, including the posterior cingulate cortex (PCC), precuneus (PCu), retrosplenial cortex (RSP), as well as the lingual gyrus (LG). The reported group difference in rsFC remained significant (p-FWE = 0.021, k = 57, peak coordinates = 12, -51, 03) after removing participants who met the clinical threshold on the SDQ total score (*i.e*., scores equal or above 17; removed MT = 4 and NMT = 3) and had missing SDQ data (removed MT =2 and NMT = 2).

A post-hoc ROI-to-ROI analysis was also performed to explore if the identified pattern of increased DMN rsFC encompasses other subsystems of this network. The analysis indicated that the MT group showed a widespread pattern of increased rsFC among DMN nodes (Fig. **S1**, Supplementary Information, Appendix SI.5), including increased connectivity between the mPFC and both right [t(53) = 3.02, p-FDR = 0.013)] and left hippocampi [t(53) = 2.77, p-FDR = 0.014)], and between the left hippocampus and the PCC [t(53) = 3.41, p-FDR = 0.004)] and PCu [t(53) = 3.67, p-FDR = 0.002)]. Moreover, the pattern of increased connectivity between the RSP with the left hippocampus [t(53) = 2.26, p-FDR = 0.058)] and with the mPFC [t(53) = 2.19, p-FDR = 0.0^58^] approached statistical significance.

### 3.4 Resting-state Functional Connectivity and Interpersonal Problem-solving Skills

**Fig. (3):**
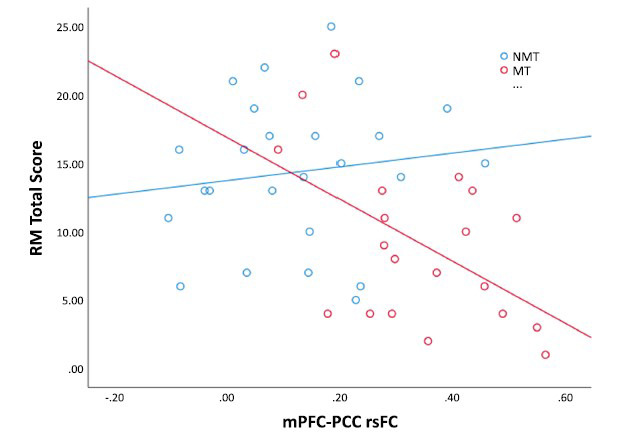
Relevant Means (RM) Total Scores are Plotted as a Function of the mPFC-PCC Resting State Functional Connectivity (rsFC) Estimates and Maltreatment Status (MT and NMT groups).

To test the hypothesis that group differences in interpersonal problem-solving are associated with maltreatment-related alterations in functional connectivity, we explored if mPFC-PCC rsFC moderates the relationships between maltreatment status and relevant means total score (MT = 20; NMT =25). The interaction term (mPFC-PCC rsFC x maltreatment status) significantly moderated relevant means total scores [B_standardized_ = -0.80 t (41) = -2.35, *p* = 0.0^2^]. Specifically, adolescents with heightened mPFC-PCC rsFC who also experienced childhood maltreatment had significantly lower RM total scores (Fig. **3**). The full regression model is reported in the Supplementary Information document, Appendix SI.6, Table **S3**.

## 4 DISCUSSION

This study examined the impact of childhood maltreatment on interpersonal problem-solving and brain functional connectivity during rest in the context of mental health functioning. There were four main findings. First, adolescents with a history of abuse and neglect, compared to non-maltreated peers, showed reduced interpersonal problem-solving performance in a vignette-based task. They generated fewer relevant means (indicating poorer means-end thinking) and less effective strategies (Table **3**). Second, impaired interpersonal problem-solving among adolescents who have experienced early adversity partly accounted for their elevated mental health symptoms (Fig. **1**). Third, young people exposed to childhood maltreatment showed increased coupling between primary and secondary DMN hubs compared to non-maltreated peers. Seed-based analyses indicated a pattern of heightened positive rsFC between the mPFC and a posterior medial cluster, encompassing the PCC, PCu, RSP, and LG (Fig. **2**). ROI-to-ROI analyses further confirmed a widespread increased rsFC between several DMN hubs, including the hippocampi, mPFC, PCC, PCu, and RSP (Appendix SI.5, Fig. **S1**). Fourth, heightened DMN rsFC partly accounted for reduced interpersonal problem-solving among adolescents who experienced childhood abuse and neglect.

Impaired social functioning is a critical mediator for the well-established link between maltreatment exposure and subsequent psychiatric risk *e.g*., [5, 44]. However, we lack a mechanistic understanding of how maltreatment-related neurocognitive alterations may lead to maladaptive social outcomes. Indirect evidence suggests that exposure to abusive or neglectful caregiving can impact problem-solving skills. Neurocognitive processes and psychological domains associated with interpersonal problem-solving skills, such as autobiographical memory [28, 29, 31], ruminative thinking [28, 38], self-efficacy [79], and social attributions and mentalizing [35-37], show atypical development among individuals exposed to early adversity [11, 13, 16, 17, 38]. Here, we establish for the first time a direct link between substantiated exposure to childhood maltreatment and impaired means-end thinking, a critical cognitive aspect of interpersonal problem-solving.

Among a wide range of individuals (not selected based on maltreatment status), poor interpersonal problem-solving has been linked with difficulties in social functioning and maladaptive psychiatric outcomes [26-33]. Thus, poorer interpersonal problem-solving skills among adolescents exposed to maltreatment and its association with elevated symptoms shed new light on how psychiatric vulnerability may become instantiated (and socially mediated) in this group of young people. Impaired or biased social-information processing, including problem-solving, may increase the likelihood of conflict (*i.e*., stress generation). It may also compromise an individual’s ability to form or maintain supportive networks (*i.e*., social thinning), a key determinant of resilient outcomes following adversity [19, 20]. On the other hand, interventions that explicitly aim to improve problem-solving skills have been found to be effective in promoting social competence and mental health in different populations (*e.g*., clinical and non-clinical) and contexts (*e.g*., primary care, school, and preschool settings) [80-83]. The findings of this study are consistent with the notion that maltreatment-related neurocognitive alterations increase psychiatric vulnerability by influencing an individual’s social architecture over time [4, 19]. Conversely, enhancing poor problem-solving represents a promising target to foster resilience *via* the development of adaptive interpersonal skills.

The DMN supports a range of neurocognitive domains relevant to social functioning, including autobiographical memory, episodic future thinking, mental scene construction, mentalizing, mind-wandering, and rumination [41]. Neurobiological models of psychopathology and extant data indicate that DMN alterations play a critical role in the pathogenesis of psychiatric disorders [44, 84]. In this study, sensitivity analyses showed that heightened DMN coupling among adolescents exposed to early adversity is evident even in the absence of overt psychopathology; yet, it is consistent with perturbations reported in meta-analytic data of individuals presenting with a frank mood disorder [48]. DMN hyperconnectivity following early abuse and neglect may therefore represent a latent psychiatric risk marker. Moreover, the maltreatment-related pattern of increased mPFC-PCC coupling (and its link with impaired interpersonal problem-solving) is highly consistent with meta-analytic data that has linked this neural signature with increased rumination [50]. Ruminative thinking interferes with problem-solving and is associated with childhood trauma and internalizing disorders commonly associated with maltreatment [38]. In sum, the pattern of increased DMN coupling following early adversity may underpin neurocognitive vulnerabilities, such as rumination, that instantiate psychiatric risk by interfering with critical aspects of social cognition, including interpersonal problem-solving skills.

This current study has a number of limitations. First, the cross-sectional design of this study does not allow us to evaluate the prognostic value of the current findings. Longitudinal designs are needed to explore if atypical DMN connectivity and problem-solving skills following maltreatment exposure contribute to future psychopathology and poor long-term social functioning. Second, it is not possible to rule out that the current findings are, at least in part, linked to neurocognitive vulnerabilities predating maltreatment exposure, known to contribute to problem-solving skills [85], such as impairments in executive functioning [86]. However, the comparable IQ and verbal fluency scores among participants with and without maltreatment exposure suggest that this is unlikely. However, prospective or genetically informed studies are required to disentangle the possible role of pre-existing factors. Third, we were statistically underpowered to use all variables of interest (*i.e*., maltreatment status, DMN rsFC, interpersonal problem-solving, and mental health symptoms) in one statistical model (*e.g*., moderated mediation) due to a reduced number of participants with available data across all domains. Future studies with larger samples (Supplementary Information, Appendix SI.7) are required to explore the presence of conditional indirect effects. Fourth, in a study with a larger sample, it would be interesting to investigate what factors may contribute to compromised interpersonal problem-solving skills and examine with more granularity the components of social and psychiatric functioning affected by maltreatment.

Several strengths also characterize this study. First, recruiting a group of young people with substantiated experiences of abuse and neglect and well-matched peers not exposed to maltreatment allows us to disentangle the effect of adverse early experiences from potentially confounding demographic variables (*e.g*., socioeconomic status, ethnicity, IQ, and verbal performance). Second, this study focuses on interpersonal problem-solving skills and DMN connectivity, two domains that, despite their centrality in the study of psychopathology and social functioning, have received scarce attention within the childhood maltreatment literature. Thus, the current study further increases our understanding of the sequelae associated with early adversity at multiple levels of explanation. Third, implementing a well-validated and reliable vignette-based measure of means-end thinking (*i.e*., the MEPS) [23, 57, 87] has allowed us to objectively assess interpersonal problem-solving skills compared to other commonly used subjective reports [88]. Fourth, findings of neurocognitive changes often account for a relatively small proportion of maltreatment-related variance (or group differences) or possess limited translational value. On the other hand, we found a statistically significant and large group difference in interpersonal problem-solving skills, which was also linked with poor mental health symptoms among adolescents exposed to early adversity.

## CONCLUSION

We investigated interpersonal problem-solving, resting-state functional connectivity (rsFC), and mental health symptoms in adolescents with maltreatment experience. Compromised interpersonal problem-solving was associated with maltreatment exposure and mental health symptomatology. Moreover, poor interpersonal problem-solving was partly accounted for by heightened coupling between DMN medial hubs, a neural signature of depression and ruminative thinking. Future studies using a longitudinal design alongside measures of social adjustment are required to explore further DMN rsFC and interpersonal problem-solving and their role in shaping social functioning and mental health outcomes after early adversity. Interpersonal problem-solving and several of its associated cognitive domains (such as rumination and autobiographical memory) are amenable to change [38, 81, 89] and are core treatment targets in evidence-based treatment protocols for a range of mental health difficulties [81, 90-92]. The current findings are consistent with interpersonal problem-solving as a promising target for preventative strategies to reduce psychiatric vulnerability and enhance resilient social functioning in adolescents with maltreatment experience.

## Figures and Tables

**Table 1:** Demographics, cognitive abilities, and symptomatology of MT (n = 40) and NMT (n = 42) Participants.

**Measures**	**MT**	**NMT**
	**N (%)**	**N (%)**
Female	19 (47.5%)	26 (61.9%)
Caucasian	26 (65%)	23 (54.8%)
SES - level of education of the parent (% beyond secondary)^a^	14 (38.9%)	23 (56.1%)
	**Mean (SD)**	**Mean (SD)**
Age	14.4 (1.6)	14.9 (1.3)
PDS - self-report^b^	2.9 (0.7)	3.1 (0.5)
WASI-IQ	104.4 (14.3)	109.1 (11.5)
Verbal fluency^c^	36.9 (10.3)	36.6 (7.6)
SDQ total score^d^	10.9 (7.4)	7.8 (6.3)
Emotional symptoms	2.8 (2.4)	2.1 (2.2)
Conduct problem	2.1 (2.0)	1.7 (2.1)
Hyperactivity/inattention*	4.0 (3.0)	2.7 (2.3)
Peer relationship problems	2.0 (1.9)	1.4 (1.5)
Prosocial behavior	7.7 (2.1)	7.7 (2.3)

**Abbreviations:** MT = Maltreated group; NMT = Non-maltreated group; PDS = Puberty Development Scale (51); SES = Socioeconomic-status; SDQ = Strength and Difficulties Questionnaire - parent report; WASI-IQ = 2 IQ-subscales derived from the Wechsler Abbreviated Scales of Intelligence.

*Participants with available data:* ^a^MT=36, NMT=41; ^b^MT=35, NMT=37; ^c^MT=36, NMT=40; ^d^MT=35, NMT=38.

* p < .05 (one-tailed).

**Table 2:** Abuse subtype, severity, and duration (in years) in the MT group (n = 40).

**Abuse Subtype**		**Mean**	**SD**
Physical abuse (*n =* 6)	Severity (0-4)	1	0
	Mean duration	5.2	5.3
Neglect (*n =* 30)	Severity (0-4)	2.4	1.8
	Mean duration	5.5	5.1
Sexual abuse (*n* = 4)	Severity (0-4)	1.8	1.5
	Mean duration	1.9	2.5
Emotional abuse (*n* = 39)	Severity (0-4)	2.9	0.9
	Mean duration	6.8	4.9
Domestic Violence (*n =* 23)	Severity (0-4)	1.9	1.1
	Mean duration	4.7	3.0

**Table 3:** Mean MEPS scores in the MT and NMT groups.

-	**MT**		**NMT**		***p^a^***	***Cohen’s d***
	**Mean**	***SD***	**Mean**	***SD***		
RM Total Score	2.11	1.55	3.61	1.67	*<.001*	*.93*
Active RM	1.76	1.27	2.70	1.18	*.001*	*.78*
Passive RM	.27	.38	.56	.70	*.02*	*.51*
No-means	1.57	1.25	1.44	2.10	*.37*	*.08*
Effectiveness	2.65	1.17	3.80	1.33	*<.001*	*.93*

**Abbreviations:** MT = Maltreated group (n = 34); NMT = Non-maltreated group (n = 38); MEPS = Means-end Problem-Solving; RM = Relevant Means. ^a^One-tailed *p*-values.

## Data Availability

The data associated with this study has yet to be made publicly accessible. (To discuss access, please contact the principal investigator, professor Eamon McCrory, e.mccrory@ucl.ac.uk).

## LIST OF ABBREVIATIONS

BOLD: Blood Oxygen Level-dependent
CSF: Cerebrospinal Fluid
DMN: Default Mode Network
EPI: Echo-planar Image
FDR: False Discovery Rate
fMRI: Functional Magnetic Resonance Imaging
FoV: Field of View
FWE: Family-wise Error
FWHM: Full-width Half Maximum
IQ: Intelligence Quotient
LG: Lingual Gyrus
MEPS: Means-end Problem Solving Task
mPFC: Medial Prefrontal Cortex
MPRAGE: Magnetization-prepared Rapid Gradient-echo Sequence
MT: Maltreatment Group
NMT: Non-maltreatment Group
PCC: Posterior Cingulate Cortex
PCu: Precuneus
ROI: Region of Interest
RSC: Retrosplenial Cortex
rsFC: Resting-state Functional Connectivity
SDQ: Strengths and Difficulties Questionnaire
SES: Socioeconomic Status
TE: Echo Time
TR: Repetition Time
