# Supplementary material for: Atypical Interpersonal Problem-Solving and Resting-state Functional Connectivity in Adolescents with Maltreatment Experience
Source: Curr Neuropharmacol. 2023 Oct 5;22(2):290–301. doi: 10.2174/1570159X22666231002145440 (PMC10788892; doi:10.2174/1570159X22666231002145440)
Supplement: Supplementary file 1 [file CN-22-290_SD1.pdf]

Supplementary Material

Atypical Interpersonal Problem-Solving and Resting-state Functional Connectivity in Adolescents with Maltreatment Experience

Mattia I. Gerin[1,2,*], Essi Viding[1], Vanessa B. Puetz[1,2], Diana J.N. Armbruster-Genc[1], Georgia Rankin[1] and Eamon J. McCrory[1,2]

[1]Division of Psychology and Language Sciences, University College London, London, UK; [2]Anna Freud National Centre for Children and Families, London, UK

Appendix SI.1 - Outliers

To remove outliers for normally distributed outcome variables Tukey's box-plot Inter-quartile range (IQR) method was implemented, using a multiplier of 2.2, as suggested by simulation estimates by (Hoaglin & Iglewicz, 1987). For non-normally distributed data the 'adjusted boxplot' method developed by (Hubert & Vandervieren, 2008) was implemented instead (Seo, 2006). One participant from the MT group was removed from MEPS analyses.

Appendix SI.2 - Resting-State Functional Connectivity (rsFC) Post-hoc ROI-to-ROI DMN analyses.

Post-hoc ROI-to-ROI analyses of the DMN were performed using the four main DMN nodes that were taken from the DMN specific masks included in the CONN toolbox that were generated from the Human Connectome Project (n=497) (Little et al., 2018; Whitfield-Gabrieli & Nieto-Castanon, 2012). These include the mPFC, PCC, and the left and right AG. Secondary hubs, such as the precuneus and left and right hippocampi (Buckner & DiNicola, 2019; Kaboodvand et al., 2018) were taken from the CONN atlas based on the FSL Oxford-Harvard atlas. The retrosplenial cortex (Kaboodvand et al., 2018) was created by adding the bilateral Broadman area 29 and 30 (Vann et al., 2009), based on the Broadman Areas atlas provided with the WFU Pickatlas toolbox (v3.0.5b) for SPM/Matlab (Friston et al., 2006; Maldjian et al., 2003). Between subject contrast analyses were performed using the CONN standard settings for ROI-to-ROI functional connectivity analyses (Nieto-Castanon, 2020; Whitfield-Gabrieli & Nieto-Castanon, 2012). The analyses consisted of 28 connections among the 8 ROIs mentioned above (mPFC, PCC, right and left AG, right and left hippocampi, precuneus, and retrosplenial cortex). Parametric multivariate statistics using cluster-level inferences were selected, with an initial cluster threshold set a $p<.05$ p-FDR corrected (MVPA omnibus F-test for dimensionality reduction of the connectivity patterns across all subjects and all the seed ROIs simultaneously), and the connection threshold was set at $p<.05$ p-FDR corrected (to make inference about individual connections using t-test).

1875-6190/24 $65.00+.00 © 2024 Bentham Science Publishers

Appendix SI.3 - Means-end Problem Solving (MEPS) task

Vignettes and task procedure

Originally the MEPS consisted of 10 vignettes (Platt & Spivack, 1975a), but subsequent validation studies (Platt & Spivack, 1975b) have indicated that it is possible to reduce participant burden and still obtain reliable data by administering 3-5 vignettes (D'Zurilla et al., 2004; Glazebrook et al., 2015; Hawton et al., 1999; Lyubomirsky & Nolen-Hoeksema, 1995; Marx et al., 1992; Oldershaw et al., 2009; Quiñones et al., 2015; Watkins & Moulds, 2005; Werner-Seidler et al., 2018). In this study, we also implemented a shortened/modified version of the original MEPS – we administered four interpersonal vignettes related to problems in different social scenarios, including peers/friends, a teacher, moving to a new neighbourhood, and leadership/school involvement. Note that, in order to increase the relevance of the problematic situations to our child/adolescent sample, two vignettes were modified from the original ones: The vignette describing a problem with a teacher was adapted from the original MEPS vignette depicting a problem with a boss/manager at work, and the vignette about the school meeting was adapted from the original MEPS vignette describing a local community meeting.

The four vignettes were:

You notice that your friends seem to be avoiding you. You want to have friends and be liked. The story ends when your friends like you again.

You had just moved into a new neighbourhood. You want to have friends in the new neighbourhood. The story ends with you having many good friends and feeling at home in the neighbourhood.

You are having trouble getting along with one of your teachers. You are very unhappy about this. The story ends with the teacher liking you.

You are listening to a group of school friends speak about how to make things better in your school. You wanted to say something important and have a chance to be your class representative. The story ends with you being elected leader and presenting a speech.

The presentation order of the vignettes was pseudorandomised, and for each scenario, participants had 2 minutes to verbally generate solutions. The vignette describing the interpersonal problem and the resolution

was always in front of the participants as they described the solution path. Before the beginning of the task, participants had one practice trial in which a non-interpersonal problematic situation was presented:

You came home after school and found that you had lost your phone. You are very upset about it. The story ends with you finding your phone and you feeling good about it.

The instructions were similar to those implemented in previous studies with adolescents and adults (Glazebrook et al., 2015; Hawton et al., 1999; Oldershaw et al., 2009; Raes et al., 2005; Williams et al., 2005). Participants imagined themselves in the problematic scenarios and generated verbally a detailed *step-by-step* strategy. During the practice trial, participants received online feedback on their performance and were reminded of the importance of generating a detailed step-by-step solution. Conversely, during the actual task, feedback was not provided, and only minimal/general prompting was allowed (e.g., "is there anything else you would like to add"). Responses were audio-recorded and then transcribed.

Scoring

In line with standard scoring procedures implemented in studies with adults and adolescents, we scored the total number of *Relevant Means*. These are defined as the discrete and concrete steps taken from the beginning of the story that brings the participant closer to the problem resolution (Glazebrook et al., 2015; Hawton et al., 1999; Kremers et al., 2006; Marx et al., 1992; Oldershaw et al., 2009; Platt & Spivack, 1975a; Raes et al., 2005). The definition of Relevant Means encompasses also: i) the acknowledgment of potential obstacles; ii) recognising that solving problems can require time; iii) the generation of alternative strategies; and iv) introspective/reflective comments (e.g., realising, deciding, wondering) (Kremers et al., 2006; Williams et al., 2005). The total number of Relevant Means generated across the four vignettes was averaged to generate an individual Relevant Means total score. Also, in line with previous studies, Relevant Means were broken down into 'Active' (these are relevant steps initiated by the participant) and *'Passive'* (these are relevant steps initiated by another person) (Kremers et al., 2006; Oldershaw et al., 2009). Steps lacking detail (e.g., 'we sorted things out') with no explanation of how things were resolved, repetitive information, and descriptions that were irrelevant to the resolution of the problem were not considered as Relevant Means; instead, these were scored as '*No-Means'* in line with current scoring procedures (Oldershaw et al., 2009). Also, in line with recent studies, we included an additional *Effectiveness* score. This was rated on a 7-point scale by the experimenter (1 = not at all effective; 7 = extremely effective). A problem-solving strategy is considered to be effective if it maximises

positive outcomes and minimises short- and long-term undesirable consequences, both to oneself and others (D’Zurilla et al., 2004; D’Zurilla & Goldfried, 1971; Kremers et al., 2006; Oldershaw et al., 2009; Raes et al., 2005; Ridout et al., 2015).

Scoring was carried out blindly (i.e., group/participant’s identity was unknown by the rater). Moreover, a second independent blind rater (a trained research assistant) scored a random subset of interviews (16%) in order to determine inter-rater reliability. Intra-class correlations (ICC) for RM total scores were calculated using a two-way random absolute agreement model. A good degree of reliability was found – the ICC was .64 [95% CIs LL = .43 UL = .79, $F(43,43) = 4.61$, $p < .001$]. Moreover, for Active RM and Passive RM, an excellent degree of reliability was found. The respective ICC was .81 [95% CIs LL = .67 UL = .89, $F(43,43) = 9.40$, $p<.001$] and .76 [95 % CIs LL = .60 UL = .86, $F(43,43) = 7.24$, $p < .001$]. For No-Means, a fair degree of reliability was found with an ICC of .50 [95% CIs LL = .25 UL = .70, $F(43,43) = 3.03$, $p < .001$]. Finally, reliability was good for Effectiveness scores – ICC was .62 [95% CIs LL = .41 UL = .78, $F(43,43) = 4.32$, $p < .001$].

Appendix SI.4 – Demographic and symptomatology of MEPS and rsFC subsamples

Table S1.
Demographics, cognitive abilities, and symptomatology of MT (n=34) and NMT (n=38) participants included in the MEPS analyses.

	MT	NMT
Measures	n (%)	n (%)
Female	16(47.1%)	22 (57.9%)
Caucasian	22(64.7%)	21(55.3%)
SES - level of education of parent (% beyond secondary)[1]	12(40.0%)	21(56.8%)
	Mean (sd)	Mean (sd)
Age	14.4 (1.7)	14.9 (1.2)
PDS - self-report[2]	2.9 (0.7)	3.1 (0.5)
WASI-IQ	104.6 (14.9)	110.1 (11.1)
Verbal fluency[3]	37.3 (10.5)	36.7 (7.7)
SDQ total score*[4]	11.3 (7.5)	7.4 (5.9)
emotional symptoms	3.0 (2.4)	2.0 (2.1)
conduct problem	2.1 (2.0)	1.5 (2.0)
hyperactivity/inattention*	4.2 (3.1)	2.6 (2.4)
peer relationship problems	2.1 (2.0)	1.3 (1.3)
prosocial behaviour	7.5 (2.2)	8.1 (1.9)

Note: * $p < .05$. *Abbreviations:* MT = Maltreated group; NMT = Non-maltreated group; PDS = Puberty Development Scale (Petersen et al., 1988); SES = Socio-economic-status; SDQ = Strength and Difficulties Questionnaire – parent report; WASI-IQ = 2 IQ-subscales derived from the Wechsler Abbreviated Scales of Intelligence. *Participants with available data:* [1] MT=30, NMT=37; [2] MT=29, NMT=34; [3] MT=34, NMT=37; [4] MT=30, NMT=35.

Table S2.
Demographics, cognitive abilities, and symptomatology of MT (n=26) and NMT (n=29) participants included in the rsFC analyses

	MT	NMT
Measures	n (%)	n (%)
Female	14 (53.8%)	18 (62.1%)
Caucasian	18 (69.2%)	15 (51.7%)
SES - level of education of parent (% beyond secondary)	10 (38.5%)	16 (55.2%)
	Mean (sd)	Mean (sd)
Age	14.6 (1.4)	15.1 (1.3)
PDS - self-report[1]	3.1 (0.6)	3.1 (0.4)
WASI-IQ	105.2 (12.7)	110.9 (10.0)
Verbal fluency[2]	36.1 (11.3)	37.5 (7.9)
SDQ total score[3]	9.3 (6.8)	7.6 (5.9)
emotional symptoms	2.6 (2.2)	2.0 (2.1)
conduct problem	2.0 (2.0)	1.6 (1.9)
hyperactivity/inattention	3.2 (2.4)	2.7 (2.4)
peer relationship problems	1.5 (1.5)	1.4 (1.6)
prosocial behaviour	8.3 (1.8)	7.6 (2.4)

Abbreviations: MT = Maltreated group; NMT = Non-maltreated group; PDS = Puberty Development Scale (Petersen et al., 1988); SES = Socio-economic-status; SDQ = Strength and Difficulties Questionnaire – parent report; WASI-IQ = 2 IQ-subscales derived from the Wechsler Abbreviated Scales of Intelligence. *Participants with available data:* [1] MT=24, NMT=26; [2] MT=22, NMT=28; [3] MT=24, NMT=27.

Appendix SI.5 – Post-hoc DMN ROI-to-ROI analyses

Figure S1

ROI-to-ROI DMN Resting State Functional Connectivity (rsFC) Differences Between Adolescents with Maltreatment History (MT) and Adolescents with No Maltreatment History (NMT)

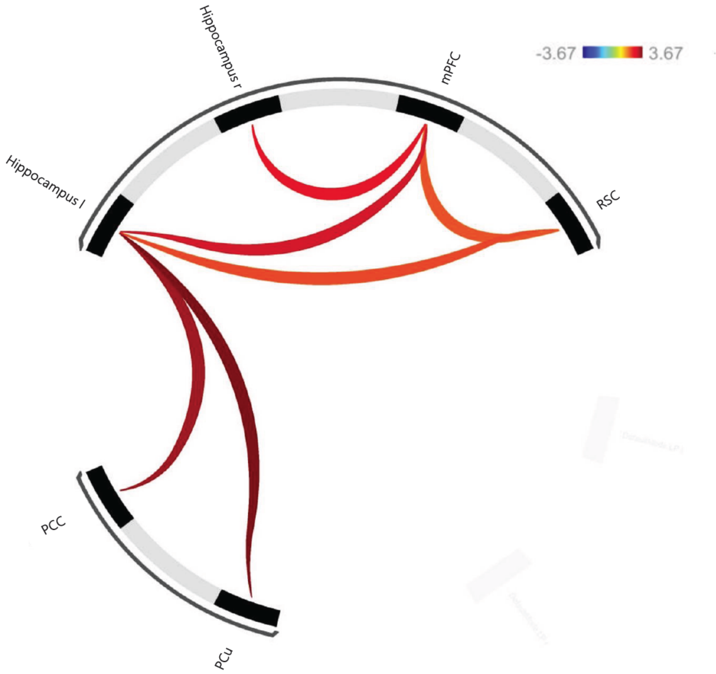

Note. Adolescents exposed to childhood maltreatment (MT) compared to peers not exposed to abuse and neglect (NMT) showed significantly increased positive resting-state functional connectivity (rsFC) between the medial prefrontal cortex (mPFC) with both right and left hippocampi, and between the left hippocampus and the posterior cingulate cortex (PCC) and precuneus (PCu). Moreover, the pattern of increased connectivity between the retrosplenial cortex (RSP) with the left hippocampus and the mPFC approached statistical significance.

Appendix SI.6 – mPFC-PCC and Maltreatment Status Moderation Results

Table S3.
Results of multiple regression predicting RM total scores for participants who had resting state and MEPS available data (MT = 20; NMT =25).

Predictor	$B_{\text{standardised}}$ or *d'*	t (41)	p
Maltreatment status	-0.48	-.1.42	.17
mPFC-PCC rsFC	0.15	0.67	.51
Interaction of Maltreatment status x mPFC-PCC rsFC *	-0.80	-2.35	.02

Note: Dependent variable = RM total score. The full model is significant $R^2 = .29$, $F(3,41) = 5.68$, $p = .002$. Significant R^2-change ($p = .02$) due to addition of interaction term = .10. * = $p < .05$. Regression coefficients are expressed as standardized Beta for continuous independent variables and *d'* for categorical independent variables. *Abbreviations:* mPFC-PCC = medial prefrontal cortex to posterior cingulate cortex cluster; rsFC = resting state functional connectivity.

Appendix SI.7 – Power Analysis for Putative Conditional Indirect Effects

The Discussion section highlights the limitations of power concerning a potential comprehensive model which includes all variables of interest (i.e., rsFC, interpersonal problem-solving, and symptom scores). We would think it useful for future studies to conduct a moderated mediation. Specifically, in our case, rsFC (the moderator W) would condition/moderate the association between maltreatment status (X) and interpersonal problem-solving performance (mediator M) and their impact on total symptoms score (Y). Thus, we have conducted a power analysis to estimate the optimum sample required for such an approach.

We used the software package for R 'simsem' to perform a power analysis for the conditional indirect effect (in a moderated mediation model based on Preacher et al. Model 2) (Donnelly et al., 2021; Preacher et al., 2007). The standardized coefficient of the b pathway in the mediation model presented in Figure 1 was $b_1 = -0.25$, and the standardized interaction/moderation coefficient of the moderation shown in Table S3 (Appendix SI.6) was $a_3 = -0.80$. Consistent with Preacher et al. (2007) Model 2 for the canonical model of moderated mediation (i.e., in which only the 'a' pathway is moderated), it would be expected that the putative standardized index of moderated mediation (also known as a conditional indirect effect) to be approximately $a_3 * b_1 = 0.20$. The outcome of a Monte Carlo Simulation estimated that future studies should recruit approximately 170 participants to test with enough statistical power (80%) the presence of a conditional indirect effect of this magnitude (i.e., 0.20). This N is also consistent with the empirical power simulations estimated for the moderated mediation of Model 2 reported in Preacher et al. (2007). In our study, only 43 participants (19 MT and 24 NMT) had usable/available data across all three variables (i.e., rsFC, interpersonal problem-solving, and SDQ symptom scores). Hence, we are underpowered to run a moderated mediation model.

Supporting Information References

Buckner, R. L., & DiNicola, L. M. (2019). The brain's default network: updated anatomy, physiology and evolving insights. In *Nature Reviews Neuroscience* (Vol. 20, Issue 10, pp. 593–608). https://doi.org/10.1038/s41583-019-0212-7

Donnelly, S., Jorgensen, T., & Rudolph, C. (2021). Power Analysis for Conditional Indirect Effects: A Tutorial for Conducting Monte Carlo Simulations with Categorical Exogenous Variables. *PsyArXiv*. https://doi.org/10.31234/OSF.IO/35768

D'Zurilla, T. J., & Goldfried, M. R. (1971). Problem solving and behavior modification. *Journal of Abnormal Psychology*, *78*(1), 107–126. https://doi.org/10.1037/h0031360

D'Zurilla, T. J., Nezu, A. M., & Maydeu-Olivares, A. (2004). Social problem solving: Theory and assessment. In E. C. Chang, T. J. D'Zurilla, & L. C. Sanna (Eds.), *Social problem solving: Theory, research, and training*. (First, pp. 11–27). American Psychological Association. https://doi.org/10.1037/10805-001

Friston, K. J., Ashburner, J., Kiebel, S., Nichols, T., & Penny, W. (2006). Statistical Parametric Mapping: The Analysis of Functional Brain Images. In *Statistical Parametric Mapping: The Analysis of Functional Brain Images*. Elsevier Ltd. https://doi.org/10.1016/B978-0-12-372560-8.X5000-1

Glazebrook, K., Townsend, E., & Sayal, K. (2015). The Role of Attachment Style in Predicting Repetition of Adolescent Self-Harm: A Longitudinal Study. *Suicide and Life-Threatening Behavior*, *45*(6), 664–678. https://doi.org/10.1111/sltb.12159

Hawton, K., Kingsbury, S., Steinhardt, K., James, a, & Fagg, J. (1999). Repetition of deliberate self-harm by adolescents: the role of psychological factors. *Journal of Adolescence*, *22*(3), 369–378. https://doi.org/10.1006/jado.1999.0228

Hoaglin, D. C., & Iglewicz, B. (1987). Fine-tuning some resistant rules for outlier labeling. *Journal of the American Statistical Association*, *82*(400), 1147–1149. https://doi.org/10.1080/01621459.1987.10478551

Hubert, M., & Vandervieren, E. (2008). An adjusted boxplot for skewed distributions. *Computational Statistics and Data Analysis*, *52*(12), 5186–5201. https://doi.org/10.1016/j.csda.2007.11.008

Kaboodvand, N., Bäckman, L., Nyberg, L., & Salami, A. (2018). The retrosplenial cortex: A memory gateway between the cortical default mode network and the medial temporal lobe. *Human Brain Mapping*, *39*(5), 2020–2034. https://doi.org/10.1002/hbm.23983

Kremers, I. P., Spinhoven, Ph., Van der Does, A. J. W., & Van Dyck, R. (2006). Social problem solving, autobiographical memory and future specificity in outpatients with borderline personality disorder. *Clinical Psychology & Psychotherapy*, *13*(2), 131–137. https://doi.org/10.1002/cpp.484

Little, G., Reynolds, J., & Beaulieu, C. (2018). Altered functional connectivity observed at rest in children and adolescents prenatally exposed to alcohol. *Brain Connectivity*, *8*(8), 503–515. https://doi.org/10.1089/brain.2017.0572

Lyubomirsky, S., & Nolen-Hoeksema, S. (1995). Effects of self-focused rumination on negative thinking and interpersonal problem solving. *Journal of Personality and Social Psychology*, *69*(1), 176–190. https://doi.org/10.1037/0022-3514.69.1.176

Maldjian, J. A., Laurienti, P. J., Kraft, R. A., & Burdette, J. H. (2003). An automated method for neuroanatomic and cytoarchitectonic atlas-based interrogation of fMRI data sets. *NeuroImage*, *19*(3). https://doi.org/10.1016/S1053-8119(03)00169-1

Marx, E. M., Williams, M. J., & Claridge, G. C. (1992). Depression and Social Problem Solving. *Journal of Abnormal Psychology*, *101*(1), 78–86. https://doi.org/10.1037/0021-843X.101.1.78

Nieto-Castanon, A. (2020). *Handbook of functional connectivity Magnetic Resonance Imaging methods in CONN* (Issue February).

Oldershaw, A., Grima, E., Jollant, F., Richards, C., Simic, M., Taylor, L., & Schmidt, U. (2009). Decision making and problem solving in adolescents who deliberately self-harm. *Psychological Medicine*, *39*(1), 95–104. https://doi.org/10.1017/S0033291708003693

Petersen, A. C., Crockett, L., Richards, M., & Boxer, A. (1988). A self-report measure of pubertal status: Reliability, validity, and initial norms. *Journal of Youth and Adolescence*, *17*(2), 117–133. https://doi.org/10.1007/BF01537962

Platt, J. J., & Spivack, G. (1975a). Manual for the Means-Ends Problem-Solving Procedure (MEPS): A measure of interpersonal cognitive problem-solving skill. In *Hanhemann Community Mental Health/Mental Retardation Center*.

Platt, J. J., & Spivack, G. (1975b). Unidimensionality of the means-ends problem-solving (MEPS) procedure. *Journal of Clinical Psychology*, *31*(1), 15–16. https://doi.org/10.1002/1097-4679(197501)31:1<15::AID-JCLP2270310106>3.0.CO;2-8

Preacher, K. J., Rucker, D. D., & Hayes, A. F. (2007). Addressing moderated mediation hypotheses: Theory, methods, and prescriptions. *Multivariate Behavioral Research*, *42*(1), 185–227. https://doi.org/10.1080/00273170701341316

Quiñones, V., Jurska, J., Fener, E., & Miranda, R. (2015). Active and passive problem solving: Moderating role in the relation between depressive symptoms and future suicidal ideation varies by suicide attempt history *Journal of Clinical Psychology*, *71*(4), 402–412. https://doi.org/10.1002/jclp.22155

Raes, F., Hermans, D., Williams, M. J., Demyttenaere, K., Sabbe, B., Pieters, G., & Eelen, P. (2005). Reduced specificity of autobiographical memory: A mediator between rumination and ineffective social problem-solving in major depression? *Journal of Affective Disorders*, *87*(2–3), 331–335. https://doi.org/10.1016/j.jad.2005.05.004

Ridout, N., Matharu, M., Sanders, E., & Wallis, D. J. (2015). The influence of eating psychopathology on autobiographical memory specificity and social problem-solving. *Psychiatry Research*, *228*(3), 295–303. https://doi.org/10.1016/j.psychres.2015.06.030

Seo, S. (2006). A review and comparison of methods for detecting outliers in univariate data sets. In *Graduate School of Public Health*. University of Pittsburgh.

Vann, S. D., Aggleton, J. P., & Maguire, E. A. (2009). What does the retrosplenial cortex do? *Nature Reviews Neuroscience*, *10*(11), 792–802. https://doi.org/10.1038/nrn2733

Watkins, E. R., & Moulds, M. (2005). Distinct modes of ruminative self-focus: Impact of abstract versus concrete rumination on problem solving in depression. *Emotion*, *5*(3), 319–328. https://doi.org/10.1037/1528-3542.5.3.319

Werner-Seidler, A., Hitchcock, C., Bevan, A., McKinnon, A., Gillard, J., Dahm, T., Chadwick, I., Panesar, I., Breakwell, L., Mueller, V., Rodrigues, E., Rees, C., Gormley, S., Schweizer, S., Watson, P., Raes, F., Jobson, L., & Dalgleish, T. (2018). A cluster randomized controlled platform trial comparing group MEmory specificity training (MEST) to group psychoeducation and supportive counselling (PSC) in the treatment of recurrent depression. *Behaviour Research and Therapy*, *105*, 1–9. https://doi.org/10.1016/j.brat.2018.03.004

Whitfield-Gabrieli, S., & Nieto-Castanon, A. (2012). Conn: A Functional Connectivity Toolbox for Correlated and Anticorrelated Brain Networks. *Brain Connectivity*, *2*(3), 125–141. https://doi.org/10.1089/BRAIN.2012.0073/ASSET/IMAGES/LARGE/FIGURE8.JPEG

Williams, M. J., Barnhofer, T., Crane, C., & Beck, A. T. (2005). Problem solving deteriorates following mood challenge in formerly depressed patients with a history of suicidal ideation. *Journal of Abnormal Psychology*, *114*(3), 421–431. https://doi.org/10.1037/0021-843X.114.3.421
